# Glucose metabolism and lncRNAs in breast cancer: Sworn friend

**DOI:** 10.1002/cam4.5265

**Published:** 2022-11-24

**Authors:** Jia‐Lin Xu, Qi Xu, Ya‐Lin Wang, Di Xu, Wen‐Xiu Xu, He‐Da Zhang, Dan‐dan Wang, Jin‐Hai Tang

**Affiliations:** ^1^ Department of General Surgery The First Affiliated Hospital of Nanjing Medical University Nanjing P.R. China; ^2^ The First Clinical School of Nanjing Medical University Nanjing P.R. China; ^3^ School of Clinical Medicine Xuzhou Medical University Xuzhou P.R. China

**Keywords:** breast cancer, glucose metabolism, lncRNA, lncRNA risk model

## Abstract

**Background:**

Glucose metabolism disorder is a common feature in cancer. Cancer cells generate much energy through anaerobic glycolysis, which promote the development of tumors. However, long non‐coding RNA may play an important role in this process. Our aim is to explore a prognostic risk model based on the glucose metabolism‐related lncRNAs which provides clues that lncRNAs predict a clinical outcome through glucose metabolism in breast cancer.

**Methods:**

1222 RNA‐seq were extracted from the TCGA database, and 74 glucose metabolism‐related genes were loaded from the GSEA website. Then, 7 glucose metabolism‐related lncRNAs risk score model was developed by univariate, Lasso, and multivariate regression analysis. The lncRNA risk model showed that high‐risk patients predict a poor clinical outcome with high reliability (P=2.838×10‐6). Univariate and multivariate independent prognostic analysis and ROC curve analysis proved that the risk score was an independent prognostic factor in breast cancer with an AUC value of 0.652. Finally, Gene set enrichment analysis showed that cell cycle‐related pathways were significantly enriched in a high‐risk group.

**Results:**

Our results showed that glucose metabolism‐related lncRNAs can affect breast cancer progression. 7 glucose metabolism‐related lncRNAs prognostic signature was established to evaluate the OS of patients with breast cancer. PICSAR, LINC00839, AP001505.1, LINC00393 were risk factors and expressed highly in the high‐risk group. A Nomogram was made based on this signature to judge patients' living conditions and prognosis.

**Conclusion:**

7 glucose metabolism‐related lncRNAs risk score model had a high prognostic value in breast cancer. PICSAR, LINC00839, AP001505.1, LINC00393 were risk factors. AP001505.1 expression was increased in most triple‐negative breast cancer cells treated with high glucose, which may also take part in breast cancer progression and potential therapeutic targets

## 1 INTRODUCTION

Breast cancer is the most frequently invasive malignancy, leading cause of cancer‐related deaths among women worldwide. The clinical cases of breast cancer account for 30% of all cancer and 15% of all cancer deaths. Molecularly, breast cancer is categorized into five subtypes (luminal A, luminal B, Her2‐overexpression, basal‐like, and normal‐like), with individual clinical characteristics and prognosis outcomes.^21^ The progression of breast cancer is closely related to pathological staging and potential molecule phenotypes.^22^ Surgery, radiotherapy, endocrine therapy, chemotherapy, and targeted molecular therapy are limited by tumor recurrence and drug resistance rates.^1^, ^6^, ^11^, ^24^ Potential biomarkers and functional targets should be applied to individualized precision therapy to lengthen the lifetime.

Glucose metabolism is consisted of three phases, glycolysis, the tricarboxylic acid cycle and oxidative phosphorylation, offering energy, and biosynthetic elements for cells.^15^ In 1924, Otto Warburg discovered the Warburg effect, who claimed that cancer cells are highly dependent on glucose metabolism by strengthening the expression level of glucose transporter proteins (GLUT1 and GLUT5) and glycolysis‐related enzyme (such as hexokinase, phosphofructokinase, and aldolase) to convert glucose into lactic acid, which promoted tumor growth, invasion, and metastasis through acid‐mediated degradation of extracellular matrix.^2^, ^4^, ^14^, ^26^, ^27^, ^32^ Founding the glucose metabolism‐related features are the feasible strategy in cancer subtype identification.

Long non‐coding RNA (lncRNA) is a kind of RNA longer than 200 nucleotides without protein translation ability.^13^ Based on the literatures on the functional mechanisms, lncRNAs are extensively documented with transcriptional regulation.^16^, ^18^ For example, lncRNA SNHG7, acting as a sponge of miR‐34a‐5p, upregulated the target gene LDHA, affecting glycolysis in breast cancer.^10^ Another study showed that *lnc00538 (YIYA)* boosted the activation of cyclin‐dependent kinase 6 (CDK6), leading to PFKFB3 phosphorylation to catalyzes F2,6BP which regulated PFK1 to enhance glycolysis.^29^ Besides, a growing number of studies have suggested that lncRNA can regulate glucose metabolism in carcinogenesis and progression.^12^, ^28^, ^30^ A sysmmetric analysis of lncRNA and glucose metabolism remains unclear.

In this article, we sifted out seven glucose metabolism‐related lncRNAs, based on RNA sequences and clinical information of breast cancer patients from TCGA, as a risk prediction model was established to evaluate patient survival and prognosis accuracy. Moreover, the co‐expression network of glucose metabolism‐related lncRNAs and mRNA showed a crucial relationship between lncRNA and glucose metabolism. Finally, the gene sets enrichment analysis implies the association of glucose metabolism—lncRNA—cell cycle‐related pathways. Our work provides a chain of evidence in this axis.

## 2 MATERIALS AND METHODS

### 2.1 Raw data's acquisition and collation

The RNA‐seq and details of the patients with breast cancer were retrieved from the TCGA (https://cancergenome.nih.gov/). 1222 RNA‐seqs and 1097 clinical information files of breast cancer are loaded from TCGA. 19,658 mRNAs and 14,124 lncRNAs were selected from the RNA‐seqs. In the clinical database, 228 patients were excluded from this study because their survival time was less than 30 days or because information were incomplete. The remaining 864 patients participated in the follow‐up survival analysis and risk scoring system.

### 2.2 Identification of glucose metabolism‐related lncRNAs

A total of 74 glucose metabolism‐related genes were selected from the Molecular Signatures Database of Gene Set Enrichment Analysis (GSEA: KEGG GLYCOLYSIS GLUCONEOGENESIS, WP GLYCOLYSIS, AND GLUCONEOGENESIS). Under using the Limma R package and Pearson correlation analysis (|Correlation Coefficient| > 0.3 and *p* < 0.001), 399 glucose metabolism‐related lncRNAs were identified by constructing the lncRNA‐mRNA co‐expression network.

### 2.3 Construction of glucose metabolism‐related lncRNA prognostic signature

The univariate Cox regression analysis was executed to search glucose metabolism‐related lncRNAs, which were significantly associated with survival (*p* < 0.01). LASSO Cox regression analysis of prognostic glucose metabolism‐related lncRNAs was performed to establish a parsimonious model, using the glmnet package in R software. Then, multivariate Cox analysis was applied to construct the optimal prognostic risk score model based on the lowest Akaike information criterion. Finally, seven glucose metabolism‐related lncRNAs were sifted out in our study. According to the formula of risk score = $\sum \text{coef}\left(\text{lncRNA}\right)\times \mathrm{Exp}\text{lncRNA}$ and the median risk score as a cut‐off point, patients were classified into high‐risk or low‐risk groups.

### 2.4 The co‐expression network between lncRNA and mRNA

The co‐expression network between lncRNA and mRNA can directly show the relationship between glucose metabolism‐related lncRNAs and their target mRNAs. The network was visualized using the Cytoscape software (version 3.6.1) and the Sankey diagram.

### 2.5 Accuracy of the risk scoring prognostic model

Through Kaplan–Meier survival curve and two‐sided log‐rank test, compare the overall survival (OS) of the high‐ and low‐risk group. Univariate and multivariate Cox regression analyses were used to evaluate whether the prognostic value of lncRNA in survival analysis was independent of other clinical variables, such as age, gender, stage, and TMN. The time‐dependent ROC (receiver operating characteristic) curves can measure the accuracy of the risk score prognostic model.

### 2.6 GSEA analysis

Gene set enrichment analysis (http://www.broadinstitute.org/gsea) was carried out based on the risk score of LncRNA into two groups. The Kyoto Encyclopedia of Genes and Genomes (KEGG) was selected to analyze significant enrichment pathway in a different group. KEGG pathways with a false discovery rate value <0.25 and *p* < 0.05 after performing 1000 permutations were considered to be significantly enriched in high/low LncRNA groups.

### 2.7 Cell culture

Human breast cancer cells (MDA‐MB‐231, BT‐549, SUM‐1315, and HCC‐1806) were obtained from the Cell Bank of the Chinese Academy of Science (Shanghai, China). In order to confirm that glucose can affect the expression levels of lncRNAs in breast cancer cells, we treated cells with high‐glucose and low‐glucose conditions. To perform a high‐glucose condition, we supplemented DMEM medium sugarless with glucose to reach DMED 20 mM glucose. Low‐glucose condition: DMEM medium sugarless with glucose to reach DMED 2 mM glucose. Cells were cultured in high‐glucose DMEM (20 mM glucose) 10% FBS and low‐glucose DMEM (2 mM glucose) 10% FBS for 24 h. Both of the cells were incubated at 37°C and 5% CO_2_ atmosphere.

### 2.8 RNA extraction and quantitative real‐time polymerase chain reaction

Reverse transcription and quantitative real‐time PCR (RT‐qPCR) were performed to affirm the sequencing results. A total of 1000 ng of RNA was reversely transcribed via using HiScript II Q RT SuperMix for qPCR (Vazyme) for cDNA synthesis, and ChamQ SYBR qPCR Master Mix (High ROX Premixed) (Vazyme) was used to perform PCR on StepOnePlus Real‐Time PCR System (Thermo Fisher Scientific). The amplification procedure was set as 95°C 30 s following 40 cycles of 95°C 10 s and 60°C 31 s. Then, the melting curve was complicated as 15 s at 95°C, 1 min at 60°C, 15 s at 95°C. The relative expressions of lncRNAs were calculated by using the 2^−ΔΔ*Ct*^ method with the internal reference being GAPDH. The following primers were used: GAPDH: 5′‐ GACTCATGACCACAGTCCATGC‐3′ (forward), 5′‐AGAGGCAGGGATGATGTTCTG‐3′ (reverse). AP001505.1F: 5′‐CGTGTTGGGAACAGCGTCC‐3′ (forward), 5′‐AAGAAACCCAGACGGTGGGA‐3′ (reverse).

### 2.9 Statistical analysis

Kaplan–Meier estimator was performed to evaluate the OS differences in patients. Univariate and multivariate cox regression were constructed to assess whether the prognostic model was independent of clinical characteristics. Furthermore, the time‐dependent ROC curves can evaluate the accuracy of the prognostic model. The above operations are implemented by using the R software package ‘Survival’ and ‘survivalROC’. All statistical analyses were carried out using R software (version 4.0.4), and *p* < 0.05 was regarded as statistically significant.

## 3 RESULTS

### 3.1 A glucose metabolism‐related lncRNA risk score model with significant prognostic value in breast cancer

**FIGURE 1:**
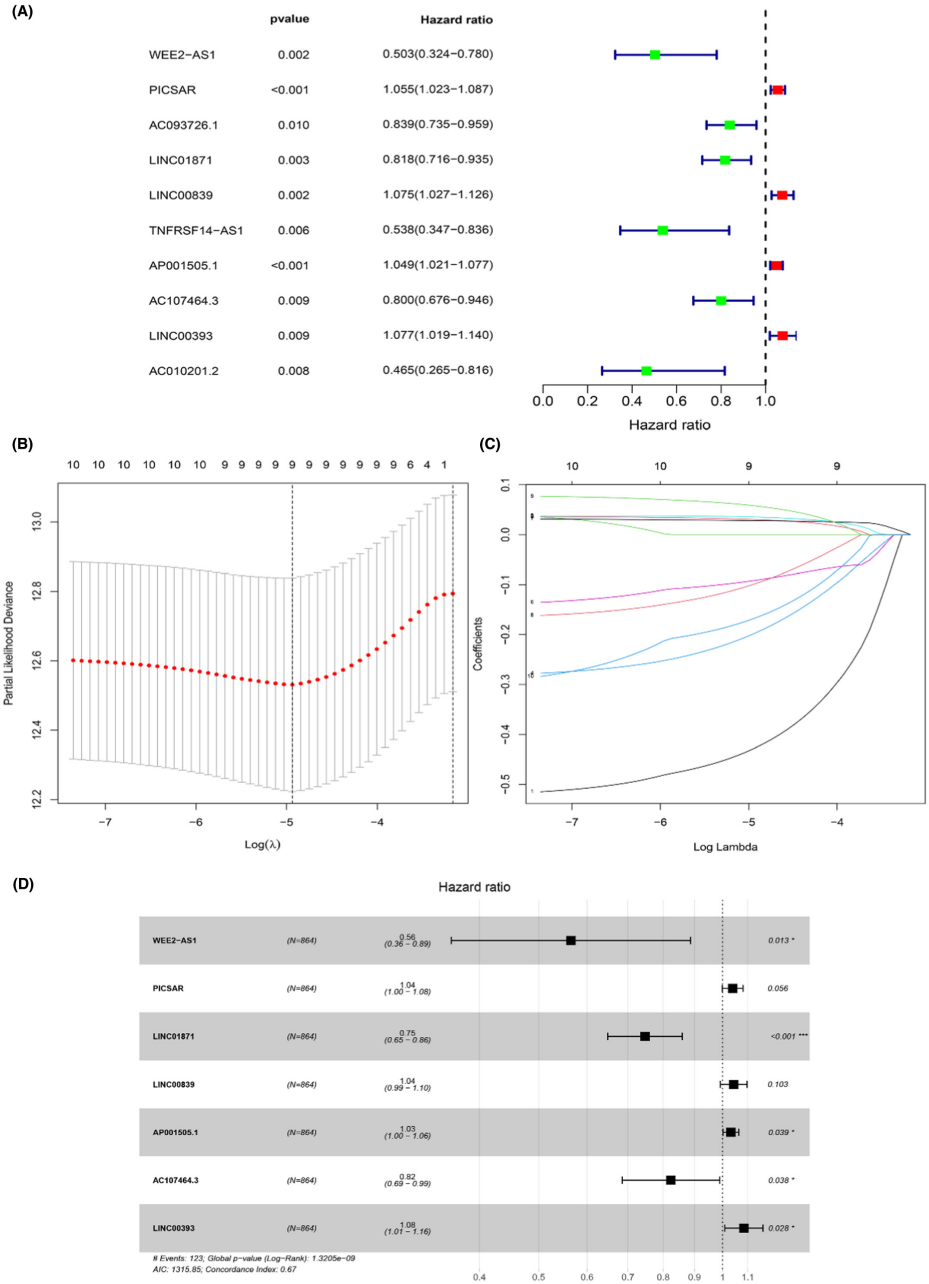
Identification of a glucose metabolism‐related lncRNA model with significant prognostic value in breast cancer. (A) The forest showed HR (95% CI) and *p*‐value of selected 10 glucose metabolism‐related lncRNAs by univariate Cox regression analysis (*p* < 0.01). (B, C) LASSO coefficient profile plot of nine variables against the log(Lambda) sequence. (D) Multivariate Cox regression analysis results show that seven glucose metabolism‐related lncRNAs correlated with overall survival of breast cancer patients. PICSAR, LINC00839, AP001505.1, LINC00393 were risk factors (hazard ratio [HR] > 1) and WEE2‐AS1, LINC01871, AC107464.3 were protective factors (HR < 1).

**FIGURE 2:**
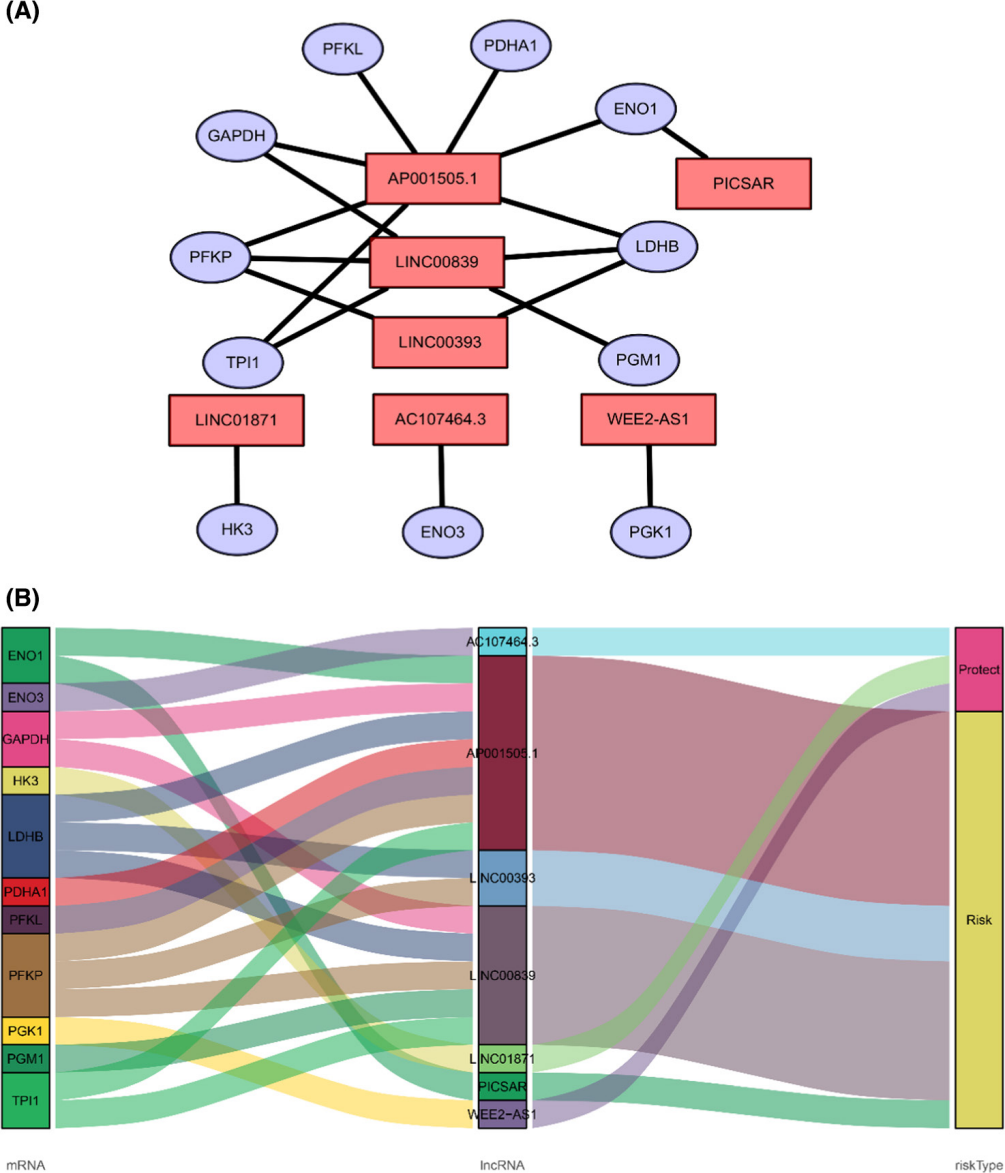
A co‐expression network of seven glucose metabolism‐related lncRNAs and glucose metabolism‐related genes. (A) The red nodes indicate lncRNAs and the purple nodes indicate glucose metabolism‐related genes. The co‐expression network is performed by Cytoscape. (B) The lncRNAs linked to shocking pink are protective lncRNAs, and dark yellow lncRNAs represent risk lncRNAs.

To determine the relationship between lncRNA and glucose metabolism, we sought out a screen glucose metabolism‐related lncRNAs in the TCGA cohort as described in “Meterials and methods” section. 74 glucose metabolism‐related genes are selected from the GSEA website. Under the screening criterion of Correlation Coefficient > 0.3 and *p* < 0.001, a co‐expression network was established between 399 glucose metabolism‐related lncRNAs and 32 glucose metabolism‐related genes. To narrow down that lncRNA into a prognostic function, univariate Cox regression analysis was carried out and showed that 10 glucose metabolism‐related lncRNAs had significant prognostic value in breast cancer patients (*p* < 0.01). Among those lncRNAs, four lncRNAs were conducive to a poor prognosis of breast cancer, which hazard ration (HR) >1 (Figure 1A). To avoid over‐fitting, the LASSO regression method was used to solve the problems of variable selection and multicollinearity. Selected nine glucose metabolism‐related lncRNAs by LASSO were used to construct the optimized prognostic signature by regulating λ in the R software “glmnet” package (Figure 1B,C). After that, multivariate Cox analysis further screened seven of nine glucose metabolism‐related lncRNAs with prognostic significance at the lowest Akaike information criterion (AIC = 1315.85), namely WEE2‐AS1 (HR = 0.564767, *p* = 0.013076), PICSAR (HR = 1.039008, *p* = 0.055939), LINC01871 (HR = 0.746676, *p* = 4.77 × 10^−5^), LINC00839 (HR = 1.042895, *p* = 0.102749), AP001505.1 (HR = 1.031788, *p* = 0.038978), AC107464.3 (HR = 0.823239, *p* = 0.037868), LINC00393 (HR = 1.084034, *p* = 0.028016) (Table 1; Figure 1D). The co‐expression network between 7 metabolism‐related lncRNAs and 11 genes was the best prognostic model. A visualized lncRNA‐mRNA coexpression network and Sankey diagram were established, which can directly identify the relationship between seven lncRNAs and 11 mRNAs by using Cytoscape software and R software (Figure 2A,B). PICSAR, LINC00893, AC110995.1, LINC00393 were risk factors (HR > 1) and WEE2‐AS1, LINC01871, AC107464.3 were protective factors (HR < 1). Then, the risk score based on the expression of seven glucose metabolism‐related lncRNAs in each patient was calculated with the formula Risk Score = (−0.57134 × the expression level of WEE2‐AS1) + (0.038266 × the expression level of PICSAR) + (−0.29212 × the expression level of LINC01871) + (0.042 × the expression level of LINC00839) + (0.031293 × the expression level of AP001505.1) + (−0.19451 × the expression level of AC107464.3) + (0.080689 × the expression level of LINC00393). Based on the median risk score, 864 patients were grouped into high‐risk (*n* = 432) and low‐risk (*n* = 432).

**TABLE 1:** The risk score prognostic model of 7 glucose metabolism‐related lncRNAs in breast cancer by multivariate Cox analysis

LncRNA	Coef	HR	HR.95L	HR.95H	*p* value
WEE2‐AS1	−0.57134	0.564767	0.359669	0.886818	**0.013076**
PICSAR	0.038266	1.039008	0.99903	1.080584	0.055939
LINC01871	−0.29212	0.746676	0.648619	0.859557	**4.77E‐05**
LINC00839	0.042	1.042895	0.991585	1.096859	0.102749
AP001505.1	0.031293	1.031788	1.001585	1.062901	**0.038978**
AC107464.3	−0.19451	0.823239	0.685148	0.989163	**0.037868**
LINC00393	0.080689	1.084034	1.008746	1.164941	**0.028016**

Abbreviations: Coef, the coefficient of lncRNAs correlated with survival; HR, hazard ratio; HR.95H, high 95% CI of HR; HR.95L, low 95%CI of HR.

*p* value less than 0.05 indicates significant difference.

### 3.2 The significance of seven glucose metabolism‐related lncRNAs prognostic model

**FIGURE 3:**
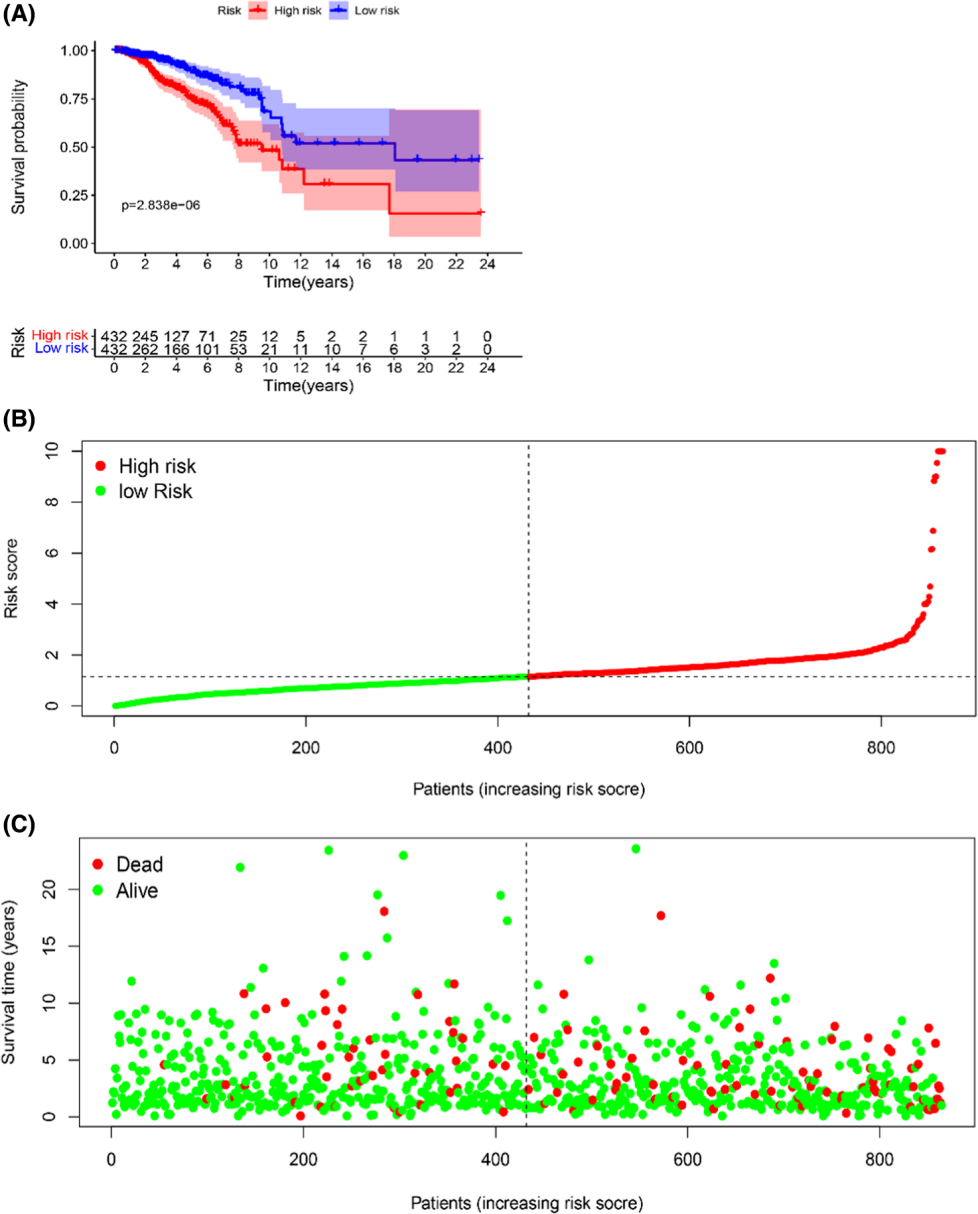
The prognostic value of seven glucose metabolism‐related lncRNAs in breast cancer. (A) Kaplan–Meier survival curve analysis of the two groups(log‐rank test, *p* = 2.306e‐09). (B) The risk curve showed the risk score of each sample. (C) The scatterplot showed the survival status of each sample. The green and red dots represent survival and death. (D) The heatmap displayed 13 glucose metabolism‐related lncRNAs expression levels in the two groups.

To further evaluate the prognostic value of the model, Kaplan–Meier survival curve analysis was performed to show a significant survival difference between the two groups (log‐rank test, *p* = 2.838 × 10^−6^, Figure 3A). The low‐risk group had a better clinical outcome than the high‐risk group, indicating a effective of the prognostic model. 864 patients were slotted from low‐risk score to high‐risk score in the Scatter plot. The patient's survival time decreased and the occurrence of deaths was boosted when the risk score was increased (Figure 3B,C).

### 3.3 Seven glucose metabolism‐related lncRNAs risk scoring system is an independent prognostic factor

**FIGURE 4:**
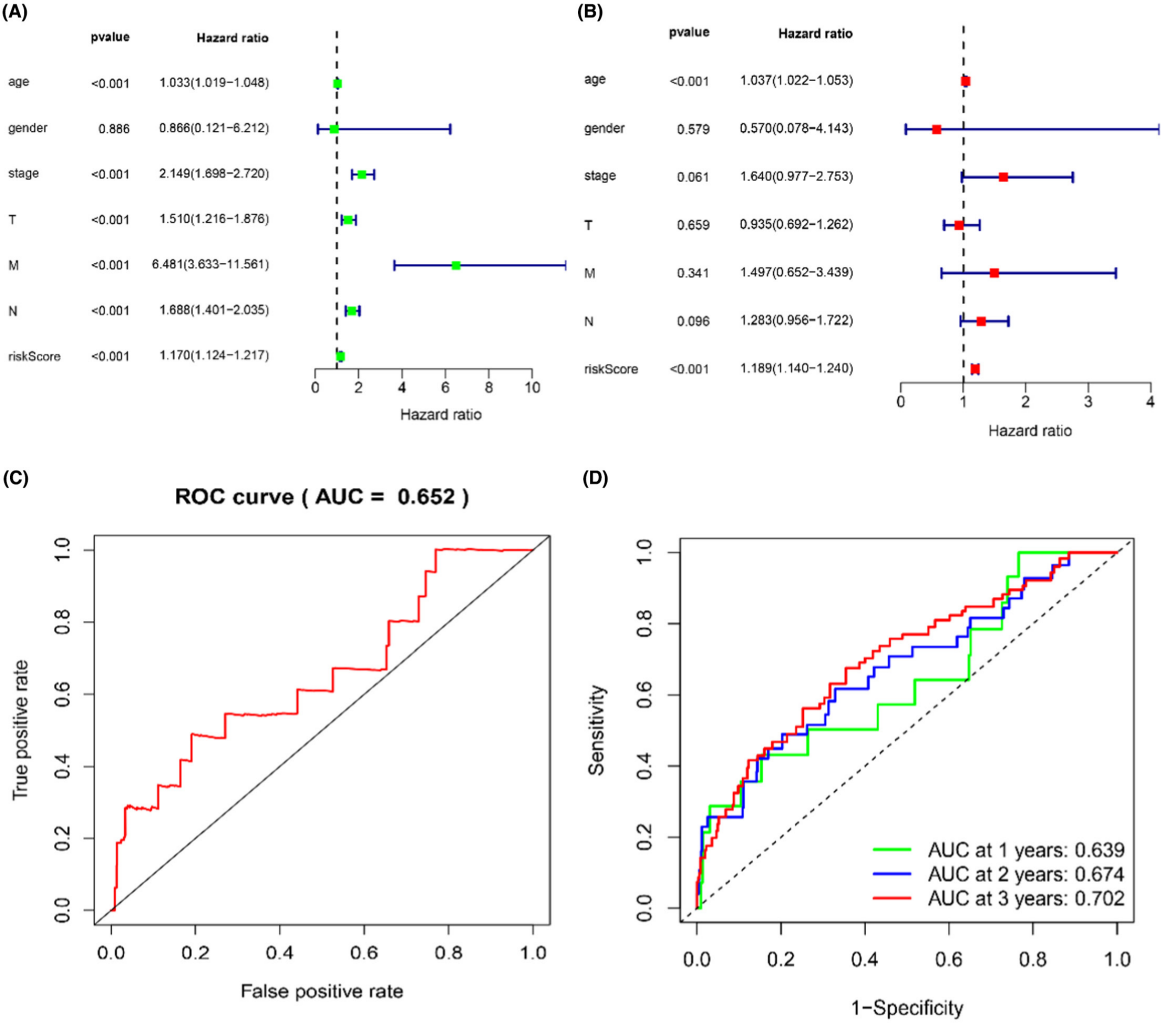
Seven glucose metabolism‐related lncRNAs risk score prognostic model is an independent prognostic factor. (A, B) Univariate and multivariate Cox regression analysis of clinical characteristics and risk score related to overall survival in breast cancer. (C) Receiver operating characteristic (ROC) curve analysis shows the accuracy of the glucose metabolism‐related lncRNAs risk score prognostic model of BC patients(AUC = 0.652). (D) Time‐dependent ROC analysis of the sensitivity and specificity of the survival for the glucose metabolism‐related lncRNAs risk score in the 1 year(AUC = 0.639), 3 years(AUC = 0.674), and 5 years (0.702).

Univariate and multivariate cox regression analyses were constructed to estimate the independent prognostic value of seven glucose metabolism‐related lncRNAs in breast cancer. Age (HR = 1.033, *p* < 0.001), stage (HR = 2.149, *p* < 0.001), T (HR = 1.510, *p* < 0.001), M (HR = 6.481, *p* < 0.001), N (HR = 1.688, *p* < 0.001), and risk score (HR = 1.070, *p* < 0.001) were significantly related with OS in univariate cox regression. However, in the multivariate cox regression, age (HR = 1.037, *p* < 0.001) and risk score (HR = 1.189, *p* < 0.001) were independent prognostic factors for OS, which indicated that 7 glucose metabolism‐related lncRNAs prognostic model was reliable (Table 2; Figure 4A,B). Additionally, the ROC curve analysis showed that the area under the ROC curve (AUC) of the risk score was 0.652 (Figure 4C). Next, we performed the AUC of the risk score at 1 year (AUC = 0.639), 2 years (AUC = 0.674), and 3 years (AUC = 0.702, Figure 4D). Those results indicated that seven glucose metabolism‐related lncRNAs signature was an independent prognostic factor with reliability and accuracy in breast cancer.

**TABLE 2:** Univariate and multivariate Cox regression analysis of clinical characteristics and risk score related to overall survival in breast cancer

Clinical features	Univariate Cox regression analyses				Multivariate Cox regression analyses			
	HR	HR.95 L	HR.95H	*p* value	HR	HR.95 L	HR.95H	*p* value
Age	1.033206	1.018509	1.048115	**7.86 × 10** ^**(−6)**^	1.037057	1.021801	1.052541	**1.49 × 10** ^**−6**^
Gender	0.865788	0.120665	6.212161	0.886025	0.569911	0.078396	4.143053	0.578522
Stage	2.149003	1.698145	2.719565	**1.92 × 10** ^**−10**^	1.640087	0.976956	2.753335	0.06124
T	1.510138	1.21557	1.876087	**0.000197**	0.934582	0.6919	1.262384	0.65918
M	6.481148	3.63338	11.56094	**2.46 × 10** ^**−10**^	1.49742	0.651995	3.439086	0.341237
N	1.688389	1.400606	2.035304	**3.94 × 10** ^**−8**^	1.283403	0.956423	1.722169	0.096311
Riskscore	1.169777	1.124158	1.217248	**1.11 × 10** ^**−14**^	1.18909	1.14012	1.240163	**6.95 × 10** ^**−16**^

*p* value less than 0.05 indicates significant difference.

### 3.4 Clinical analysis of seven glucose metabolism‐related lncRNAs model in breast cancer

**FIGURE 5:**
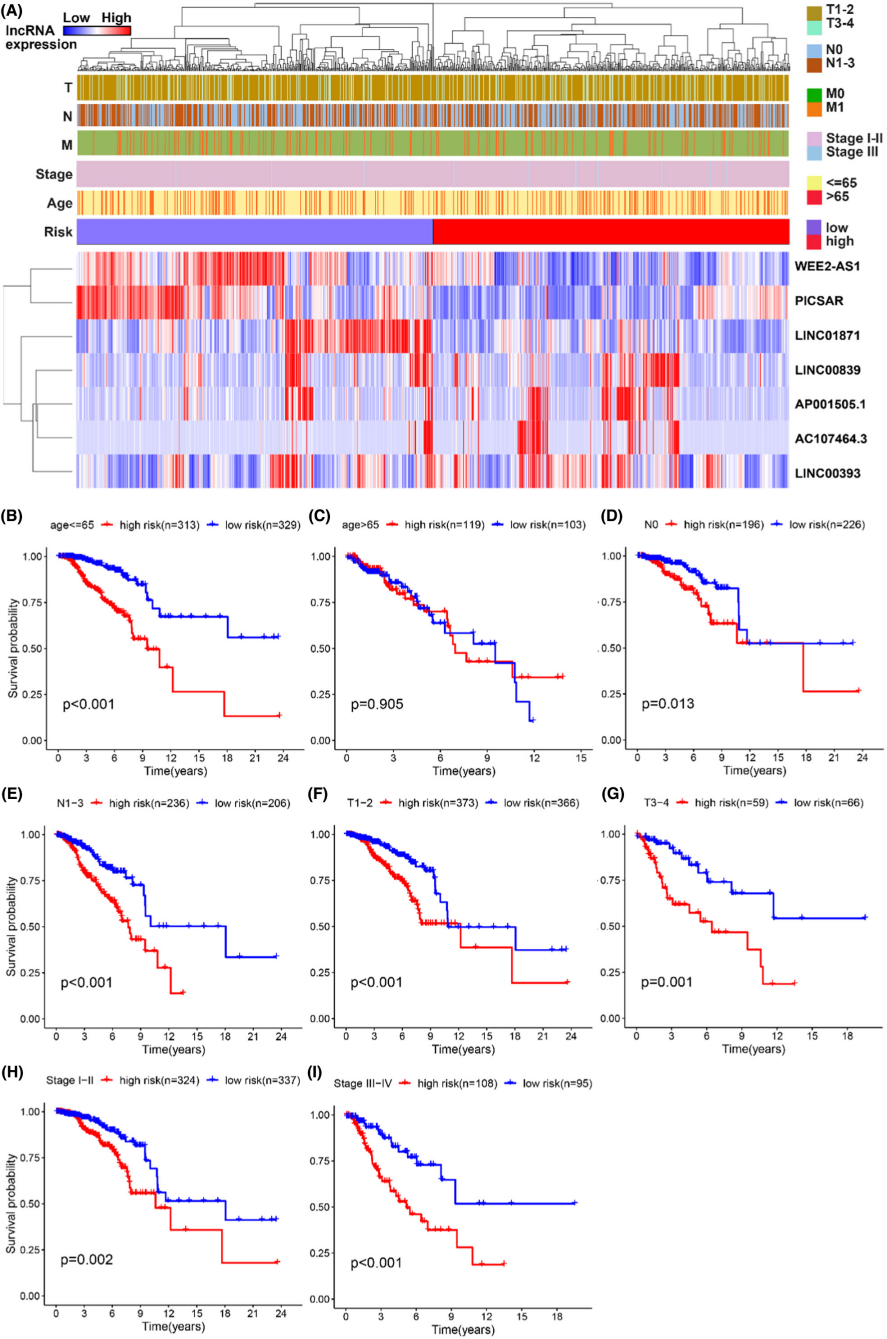
The survival probability of high‐ and low‐risk by different clinical characteristics in breast cancer. (A) The heatmap combines clinical information with the expression of seven lncRNAs and uses blue to show the low expression of lncRNA and red to show the high expression of lncRNA. PICSAR, LINC00839, AP001505.1, and LINC00393 were risk factors that can be identified directly. (B) Kaplan–Meier survival curve analysis showed survival difference between high‐ and low‐risk BC patients which were stratified by (A, B) age (>65 year vs. ≤65 year), (C, D) N (N0 vs. N1–3), (E, F) T (T1–2 vs. T3–4), (G, H) stages (stage I–II vs. stage III–IV).

To further dissect lncRNA model performance according to different clinical pathology subtypes, we divided patients into two groups according to different clinical features, such as age (≤65 and >65), gender (female and male), stage (stages I–II and stages III–IV), T stage (T1–2 and T3–4), M stage (M0 and M1) and N stage (N0 and N1–3). The heatmap combined clinical information with the expression of seven lncRNAs related to glucose metabolism. Sorted the patients by risk score, using blue to show the low expression of lncRNA and red to show the high expression of lncRNA, the expression of PICSAR, LINC00839, AP001505.1, LINC00393 with poor clinical outcomes were significant enrichment in the high‐risk group (Figure 5A). Then, We compared the survival differences of high and low‐risk groups under different clinical characteristics by using the Kaplan–Meier survival curve. We found that in the younger (≤65), no matter T and N stage (T1–2 and 3–4, N0 and 1–3, stage I–II and III–IV), the survival time of the high‐risk group was significantly shorter than that of the low‐risk group as time increased (Figure 5B–I). Those charts claimed that it was meaningful to predict patients' living conditions and prognosis value through the lncRNA risk scoring system.

**FIGURE 6:**
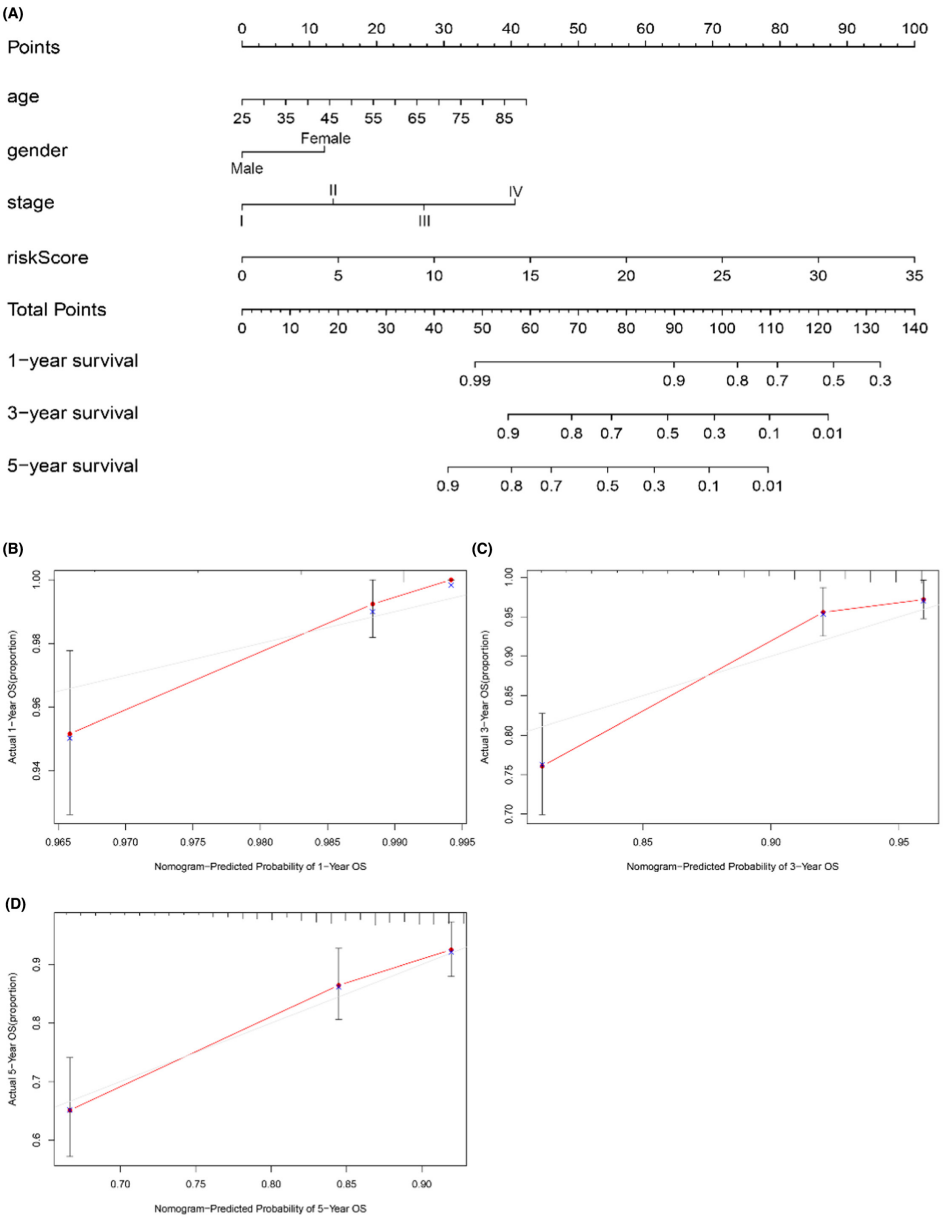
The nomogram was established to predicted 3‐, 5‐, and 10‐year overall survival in breast cancer. (A) The predicted 1‐, 3‐, 5‐year survival rates of BC patients based on the risk score from glucose metabolism‐related lncRNAs model and clinical characteristics such as age, gender, stage. (B–D) Calibration curves show the concordance between predicted and observed 1‐, 3‐, and 5‐year survival rates of high‐ and low‐risk BC patients based on the prognostic nomogram after bias correction.

We made a Nomogram, which could easily judge patients' living conditions and prognosis (Figure 6A). When a patient saw the doctor, we evaluated the patients' 1‐, 3‐, and 5‐year survival by their age, gender (female = 0, male = 1), stage, T, M, N, and riskScore. The concordance index (*C*‐index) value for the nomogram was 0.79. The calibration curve analysis showed that the predicted 1‐, 3‐, and 5‐year OS was similar to the actual 1‐, 3‐, and 5‐year OS, which indicated that the nomogram scoring method was reliable and practical (Figure 6B–D).

### 3.5 Functional pathway enrichment analysis relieved biological value in breast cancer

**FIGURE 7:**
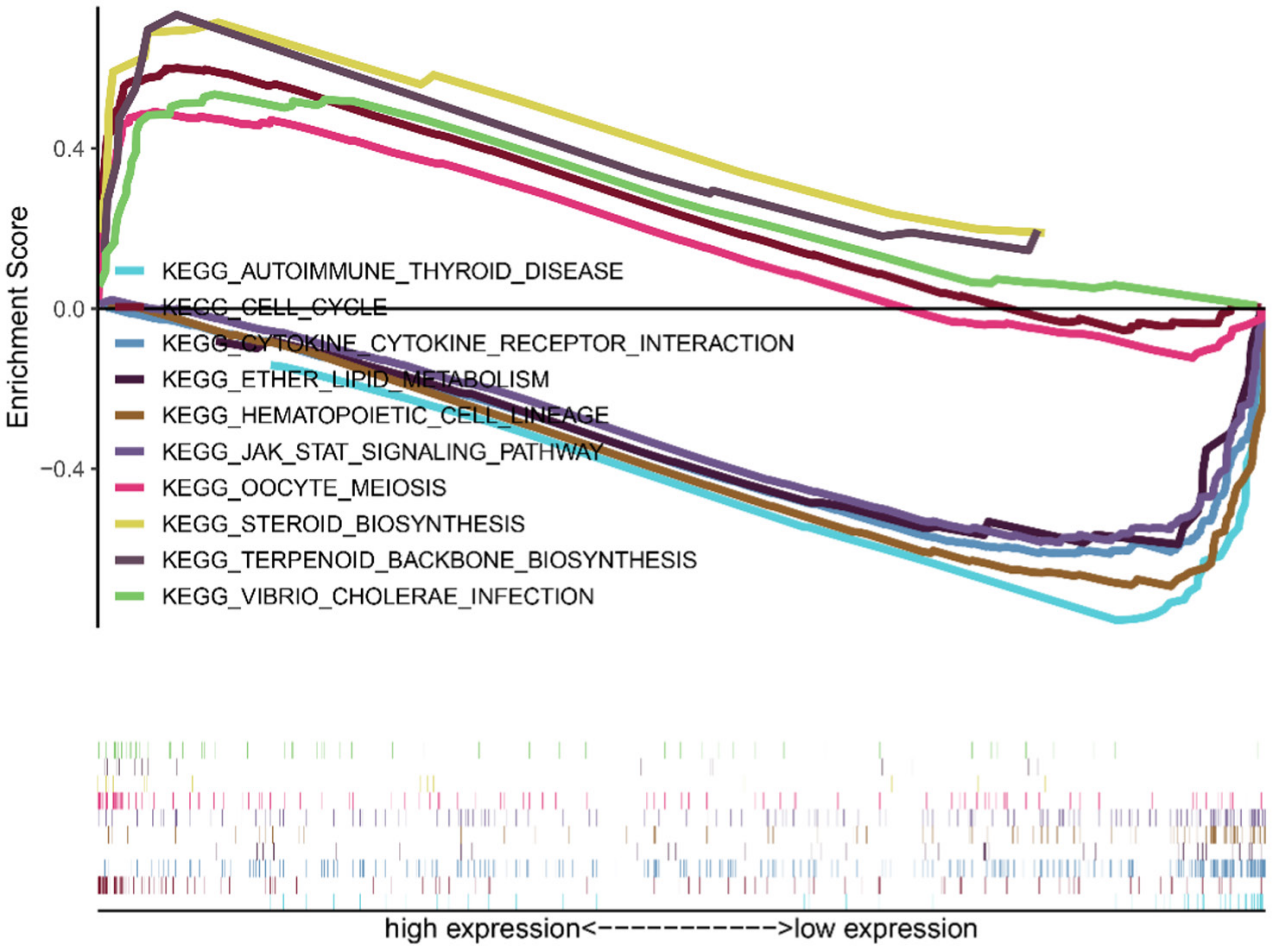
Gene Set Enrichment Analysis (GSEA) functional analysis. GSEA suggested notably enrichment pathways in the high‐risk and low‐risk groups in breast cancer.

To figure out functional pathway enrichment in high and low risk lncRNA groups, KEGG pathway analysis in Gene Set Enrichment Analysis (GSEA) was performed (Figure 7). The results showed that high risk groups statistical significance enriched in cell cycle and biosynthesis‐related pathway (cell cycle, oocyte meiosis, steroid biosynthesis); the low risk group enriched the immune response and metabolism‐related pathway (JAK–STAT signaling pathway, autoimmune thyroid disease, cytokine‐cytokine receptor interaction). These pathways may be potential therapeutic targets of breast cancer.

### 3.6 Validating the expression of AP001505.1 in Breast Cancer cells

**FIGURE 8:**
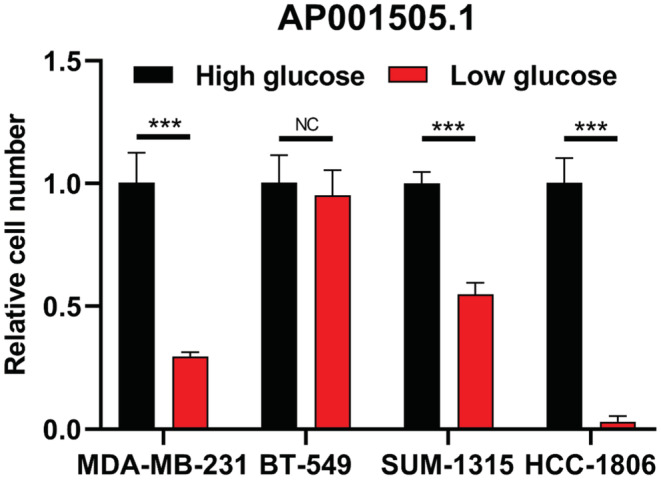
The expression of AP001505.1 in breast cancer cells. qRT‐PCR results showed that AP001505.1 expression was higher in high‐glucose condition in MDA‐ MB‐231, SUM‐1315, and HCC‐1806, and no significant changes in BT‐549. ***p < 0.001.

During the development of cancer, cells undergo glycolysis under aerobic conditions to absorb more glucose to maintain cell growth. Therefore, cancer cells are more likely to grow in high sugar environment, which is not conducive to the prognosis of cancer development. In the Risk Score prognostic model and lncRNA‐mRNA co‐expression network, AP001505.1 and LINC00839 are associated with many glucose metabolism‐related mRNAs, and also play an important role in the prognostic development of breast cancer. LINC00839 promotes chemotherapy resistance in breast cancer by targeting MYC to activate the PI3K/AKT signaling pathway. Therefore, we suspect that AP001505.1 can also affect the progression, invasion, metastasis, and recurrence of breast cancer by regulating glucose metabolism. By comparing the expression of AP001505.1 in breast cancer cells treated with high glucose and low glucose, we found that AP001505.1 expression was increased in MDA‐MB‐231, SUM‐1315, and HCC‐1806 treated with high glucose. However, BT‐549 cells showed no significant changes in high‐glucose and low‐glucose groups (Figure 8).

## 4 DISCUSSION

Glucose metabolism alternations is a hallmark of cancer cells. Glucose is the primary energy supporter, which may play a role in the process of cell proliferation and metabolism reprogramming.^19^, ^25^ The emerging evidence showed that association between lncRNAs and glucose metabolism. For example, LncRNA BCRT1 binds with miR‐1303 to prevent the degradation of its target gene PTBP3. Miranda Y.Fong et al. found that the miR‐122 secreted by breast cancer cells can inhibit the glucose uptake of non‐tumor cells in the premetastatic niche to increase cancer utilization cells and promote the metastasis of breast cancer.^9^ In another study, LINC00346 is knocked out to reduce cell proliferation and glycolysis as well as induce apoptosis through the upregulation of miR‐148a/b.^17^ Therefore, we hypothesized that glucose metabolism‐related lncRNAs were related to the recurrence, progression, metastasis, and prognosis of breast cancer. A prognosis risk model of seven glucose metabolism‐related lncRNAs was established to evaluate the clinical outcomes of patients.

In our study, 1222 RNA‐seq were extracted from the TCGA database, and 74 glucose metabolism‐related genes were loaded from the GSEA website. Through cox‐lasso regression analysis, seven glucose metabolism‐related lncRNAs were identified, which were related to the prognosis of patients. PICSAR, LINC00839, AP001505.1, LINC00393 were risk factors that promoted the breast cancer progression. Among four lncRNA, LINC00839 had been reported to promote breast cancer proliferation and chemoresistance via Lin28B‐mediated upregulation of Myc and activation of the PI3K/AKT pathway.^3^

The mechanism of lncRNAs in regulation of glucose metabolism is mainly through transcriptional and post‐transcriptional regulation through miRNAs.^20^ lncRNAs can promote the metastasis and invasion of cancer cells by regulating some critical enzymes, such as hexokinase, pyruvate kinase M, and lactate dehydrogenase, which affect glycolysis, oxidative phosphorylation, and pentose phosphate pathway.^5^, ^8^, ^23^ Several evidences supporting our risk model that lncRNA regulation glucose metabolism genes. For example, lnc00839 is reported to promote glycolysis through miRNA‐GLUT1 axis.^31^ Lnc00393 are upregulated under hyperglycemia condition in colorectal cancer.^7^ Secondly, we divided the patients into high and low‐risk groups for analysis, according to the lncRNA risk score. Kaplan–Meier survival curve analysis showed that the OS of the low‐risk group was significantly longer than that of the high‐risk population. Next, univariate and multivariate cox regression analysis showed that the risk score was an independent prognostic factor in breast cancer. Therefore, we set up a nomogram to evaluate the survival condition of the patient, such as a 50‐year‐old female patient with stage III, T_3_M_0_N_3_ and riskScore = 2.010627. Her 3‐year survival rate was 0.8–0.9, according to the nomogram (age 16 points, gender 12 points, stage III 27 points, riskScore 5 points, total points 60).

Our analysis also had many limitations, including breast cancer subtypes that were not considered for stratified analysis, and databases were not divided to improve the accuracy of the prognostic model. Secondly, patients' clinical information was incomplete and lacked immunohistochemical results (ER, PR, Her‐2, ki67), which were essential to determine the treatment of the patient. The glucose metabolism‐related genes were extracted incompletely from GSEA may affect the construction of the prognostic model. We only constructed a prognostic model by extracting breast cancer information from the TCGA database, and more experiments needed to be carried out to verify the results of bioinformatics analysis.

## 5 CONCLUSION

Seven glucose metabolism‐related lncRNAs prognostic signature was established to evaluate the OS of patients with breast cancer. PICSAR, LINC00839, AP001505.1, and LINC00393 were risk factors and expressed highly in the high‐risk group. LINC00839 has been studied in breast cancer, other lncRNAs may also take part in breast cancer progression and potential therapeutic targets.

## AUTHOR CONTRIBUTIONS

**Jia‐Lin Xu:** Data curation (lead); writing – original draft (lead). **Qi Xu:** Data curation (equal). **Ya‐Lin Wang:** Formal analysis (equal). **Di Xu:** Formal analysis (equal). **Wen‐Xiu Xu:** Methodology (equal). **He‐Da Zhang:** Visualization (equal). **Dan‐dan Wang:** Visualization (equal). **Jin‐Hai Tang:** Funding acquisition (lead).

## FUNDING INFORMATION

This research was supported by the National Key Research and Development Program of China (No. 2016YFC0905900), National Natural Science Foundation of China (No. 81872365) and Jiangsu Provincial Key Research Development Program (No. BE2019731).

## CONFLICT OF INTEREST

No potential conflict of interest was reported by the author(s).

## Data Availability

The datasets used and/or analyzed during the current study are available from the corresponding author on reasonable request (https://cancergenome.nih.gov/).
